# Upregulation of IGF2BP2 Promotes Oral Squamous Cell Carcinoma Progression That Is Related to Cell Proliferation, Metastasis and Tumor-Infiltrating Immune Cells

**DOI:** 10.3389/fonc.2022.809589

**Published:** 2022-03-01

**Authors:** Lijie Zhou, Hongyu Li, Hongshi Cai, Wenhui Liu, Enjiu Pan, Dongsheng Yu, Shuai He

**Affiliations:** ^1^ Department of Stomatology, The Second Affiliated Hospital and Yuying Children’s Hospital of Wenzhou Medical University, Wenzhou, China; ^2^ Department of Oral and Maxillofacial Surgery, Hospital of Stomatology, Guanghua School of Stomatology, Sun Yat-sen University, Guangzhou, China; ^3^ Guangdong Provincial Key Laboratory of Stomatology, Sun Yat-sen University, Guangzhou, China; ^4^ Department of Oral Emergency, Hospital of Stomatology, Guanghua School of Stomatology, Sun Yat-sen University, Guangzhou, China

**Keywords:** IGF2BP2, oral squamous cell carcinoma, prognosis, WGCNA, GSEA, immunity

## Abstract

The strong invasive and metastatic abilities of oral squamous cell carcinoma (OSCC) cells in the early stage are the main reason for its poor prognosis. The early diagnosis and treatment of OSCC may reduce the metastasis rate and improve the survival rate. The aim of this study was to explore candidate biomarkers related to the prognosis and progression of OSCC. We performed weighted gene coexpression network analysis to identify key modules and genes associated with OSCC and intersected the differentially expressed genes (DEGs) in The Cancer Genome Atlas (TCGA)-OSCC and GSE30784 datasets. Next, we performed survival analysis and immunohistochemistry to screen and validate the hub gene insulin-like growth factor 2 (IGF2) mRNA binding protein 2 IGF2BP2. We also used TCGA pan-cancer data to verify that IGF2BP2 was expressed at high levels in a variety of cancers and was related to a poor prognosis in patients. Furthermore, we divided patients with OSCC into high and low expression groups based on the median expression level of IGF2BP2. Gene set enrichment analysis (GSEA) showed that IGF2BP2 led to a poor prognosis in OSCC by affecting cancer-related (epithelial-mesenchymal transition, glycolysis, cell cycle, etc.) and immune-related biological functions and pathways. Single-sample GSEA (ssGSEA), CIBERSORT, and xCell algorithms helped reveal that high IGF2BP2 expression was accompanied by a significant reduction in the immune score, stromal score, and microenvironment score and a decrease in the number of infiltrating CD8+ T cells in OSCC. In addition, silencing IGF2BP2 suppressed the proliferation, migration, and invasion of OSCC cells. In general, IGF2BP2 is a potential biomarker for the progression, immunotherapy response, and prognosis of OSCC.

## Introduction

Oral squamous cell carcinoma (OSCC) accounts for approximately 90% of oral cancers and has a high degree of malignancy; it can rapidly invade tissues and readily form metastases in the cervical lymph nodes and distant sites (1, 2). According to global cancer statistics in 2020, more than 370,000 cases of OSCC were diagnosed, and more than 170,000 patients died due to the disease (3). Despite the existence of mature diagnosis and comprehensive treatment methods, the 5-year overall survival (OS) rate of patients with OSCC has not been significantly improved in the past few decades and is still at approximately 50% (3, 4). Therefore, it is essential to clarify the causes and mechanisms of OSCC malignant progression and to explore more effective treatment strategies.

In recent years, with the rise of high-throughput sequencing technology, a large number of omics datasets [such as those in The Cancer Genome Atlas (TCGA) database (https://www.cancer.gov/tcga)] have been generated, and the Gene Expression Omnibus (GEO) database (https://www.ncbi.nlm.nih.gov/geo/), which stores high-throughput sequencing data, emerged. Due to the rapid development of bioinformatics, key pathways and genes involved in cancer have been identified based on a biomolecular network analysis. Weighted gene coexpression network analysis (WGCNA) is a systems biology method suitable for complex multisample data analysis. It can assess the expression relationship between genes, construct a coexpression network, identify gene modules consisting of highly coexpression genes, and combine gene modules. Correlation analysis of the phenotype of the sample can reveal the module related to the phenotype (5). WGCNA can identify gene modules that are highly related to the malignant progression of OSCC to explore the genes and biological processes that have changed in OSCC patients and normal controls.

Human insulin-like growth factor 2 (IGF2) mRNA binding protein 2 (IGF2BP2/IMP2) has a molecular mass of 66 kDa, with two N-terminal RNA recognition motifs and four C-terminal human heterogeneous ribonucleoprotein-K homologous structures (6, 7). Previously, IGF2BP2 was considered to be a gene related to type 2 diabetes (T2D) (6, 8). In fact, IGF2BP2, as an RNA-binding protein, regulates cell metabolism in human metabolic diseases such as diabetes, obesity and fatty liver through the posttranscriptional regulation of many genes in a variety of cell types (9). New evidence shows that IGF2BP2 is an m6A-binding protein that promotes mRNA (for example, MYC) stability and translation in an m6A-dependent manner and participates in the development and progression of several malignant cancers (10, 11). The expression of IGF2BP2 is significantly upregulated in head and neck squamous cell carcinoma (HNSC) tissue and predicts a poor prognosis (12). In addition, IGF2BP2 polymorphisms are associated with adverse clinical features and the development of oral cancers (13).

In this study, we performed WGCNA to identify key modules and genes associated with OSCC. Next, we assessed the prognostic value of the differentially expressed genes (DEGs) to screen out the hub gene IGF2BP2. Furthermore, we divided patients with OSCC into high- and low-expression groups based on the median expression value of IGF2BP2. Gene set enrichment analysis (GSEA) was preformed to explore the biological functions related to the expression of IGF2BP2 in OSCC. Single-sample GSEA (ssGSEA), CIBERSORT, and xCell were applied to evaluate the effect of IGF2BP2 expression on immune cell infiltration and the microenvironment in OSCC. Finally, we conducted a series of functional experiments *in vitro* to investigate the impact of IGF2BP2 on cell proliferation, migration, and invasion.

## Materials and Methods

### OSCC Samples and Cell Culture

Ten OSCC tissues and their corresponding adjacent noncancerous normal tissues were collected from the Stomatology Hospital of Sun Yat-sen University with approval from the Stomatology Hospital Research Ethics Committee. All patients signed an informed consent form for participation in this study. The human OSCC cell lines SCC25 and CAL27 were obtained from the American Type Culture Collection (ATCC). SCC25 cells were cultured in Dulbecco’s modified Eagle’s medium/Ham’s F12 (DMEM/F12, Gibco, USA) supplemented with 10% fetal bovine serum (FBS, WISENT, Canada) and 400 ng/mL hydrocortisone (H811182, MACKLIN, China). CAL27 cells were grown in Dulbecco’s modified Eagle’s medium (DMEM, Gibco, USA) containing 10% FBS. Cells were cultured in a humidified incubator at 37°C and 5% CO_2_.

### Data Collection and Processing and Identification of DEGs

The mRNA expression [log2(FPKM+1)] and corresponding clinical data of the OSCC patients (providing 306 OSCC samples and 30 matched normal samples) in the TCGA were downloaded from the UCSC Xena browser (https://xenabrowser.net) (14). The mRNA expression data were converted to log2 (TPM+1) values and used for further analysis. The gene expression matrix and corresponding clinical data from the GSE30784 and GSE42743 datasets were downloaded from the GEO database. The GSE30784 dataset contains data on 167 OSCC samples and 45 normal samples. The GSE42743 dataset includes information on 74 OSCC samples and 29 normal samples. We used the R package “limma” to analyze and filter DEGs with | log2(fold change) |> 1 and false discovery rate (FDR) <0.05 (15).

### WGCNA

Genes with a standard deviation (SD) > 1 were used to construct a weighted gene coexpression network with the WGCNA package (5). First, we applied the goodSamplesGenes function to detect the quality of samples and genes and performed hierarchical clustering analysis on the samples through the average linkage method of the hclust function to screen and eliminate outliers. Second, after obtaining the best soft threshold power (β) according to the powerEstimate function, we selected a β value of 5 for TCGA-OSCC dataset and 11 for the GSE30784 dataset to construct a weighted gene coexpression network based on scale-free topology. Next, we converted the expression matrix into an adjacency matrix using the adjacency function, and then implemented the TOMsimilarity function to convert the adjacency matrix into a topological overlap matrix, and calculated the degree of dissimilarity between genes. Third, based on the degree of dissimilarity between genes derived from the topological overlap matrix, we applied the dynamic shear tree method to hierarchically cluster the genes and set the minimum number of genes in the module to 30. The gene modules with dissimilarity less than 0.2 (correlation greater than 0.8) were merged using the mergeCloseModules function. Finally, we obtained the correlation coefficients and P values of each module eigengene and trait. The module eigengene with the largest correlation coefficient and smallest P value was the hub module.

### Identification and Validation of the Hub Gene

We took the intersection of DEGs and genes in the modules most relevant to OSCC, all of which were obtained from TCGA-OSCC and GSE30784 datasets. The Venn diagram drawn with jvenn shows the intersection of these 4 gene sets and identified 12 overlapping genes (16). Univariate Cox analysis was performed to determine the correlation of the expression of these 12 genes with OS in TCGA-OSCC and GSE42743 datasets. The P value, hazard ratio (HR), and 95% confidence interval (CI) of each gene are presented in a forest plot. With a P value < 0.05 serving as the threshold, the two genes ANO1 and IGF2BP2 were finally screened out. In addition, univariate Cox analysis was applied to analyze the relationship of ANO1 expression and IGF2BP2 expression to recurrence-free survival (RFS) from TCGA-OSCC dataset. To verify the relationship between ANO1 and IGF2BP2 and the OS of HNSC patients in TCGA database using the Gene Expression Profiling Interactive Analysis 2 (GEPIA2) (http://gepia2.cancer-pku.cn/) web server.

### Pan-Cancer Expression and Prognostic Value of ANO1 and IGF2BP2

GEPIA2 is an online website containing RNA-seq and clinical data of tumor tissues and normal tissues from the TCGA and GTEx databases. The TCGA pan-cancer database has 33 cancer subtypes. The included subtypes are abbreviated as follows: ACC, adrenocortical carcinoma; BLCA, bladder urothelial carcinoma; BRCA, breast invasive carcinoma; CESC, cervical squamous cell carcinoma and endocervical adenocarcinoma; CHOL, cholangiocarcinoma; COAD, colon adenocarcinoma; DLBC, lymphoid neoplasm diffuse large B-cell lymphoma; ESCA, esophageal carcinoma; GBM, glioblastoma multiforme; HNSC, head and neck squamous cell carcinoma; KICH, kidney chromophobe; KIRC, kidney renal clear cell carcinoma; KIRP, kidney renal papillary cell carcinoma; LAML, acute myeloid leukemia; LGG, brain lower grade glioma; LIHC, liver hepatocellular carcinoma; LUAD, lung adenocarcinoma; LUSC, lung squamous cell carcinoma; MESO, mesothelioma; OV, ovarian serous cystadenocarcinoma; PAAD, pancreatic adenocarcinoma; PCPG, pheochromocytoma and paraganglioma; PRAD, prostate adenocarcinoma; READ, rectum adenocarcinoma; SARC, sarcoma; SKCM, skin cutaneous melanoma; STAD, STOMACH adenocarcinoma; TGCT, testicular germ cell tumor; THCA, thyroid carcinoma; THYM, thymoma; UCEC, uterine corpus endometrial carcinoma; UCS, uterine carcinosarcoma; and UVM, uveal melanoma. We carried out differential expression analysis and OS analysis of ANO1 and IGF2BP2 in TCGA pan-cancer datasets by using GEPIA2.

### GSEA

GSEA can be applied to analyze the enrichment of gene expression in biological functions and pathways (17). We performed GSEA between the high- and low- IGF2BP2 expression groups in TCGA-OSCC and GSE30784 datasets. The analysis of enriched biological functions and pathways according to IGF2BP2 expression was analyzed using hallmark gene sets and Kyoto Encyclopedia of Genes and Genomes (KEGG) gene sets. Gene sets with an adjusted P value < 0.05 and FDR <0.05 were considered significantly enriched.

### IGF2BP2 Expression and Immune Cell Infiltration in OSCC

We used the CIBERSORT algorithm to analyze the RNA-seq data from the OSCC samples in TCGA-OSCC and GSE30784 datasets and determined the relative proportions of 22 tumor-infiltrating immune cells in each sample (18). ssGSEA was performed using the R package “GSVA” to quantify the enrichment scores of 28 immune cells in OSCC samples in TCGA-OSCC and GSE30784 datasets (19). In addition, we employed xCell (20), a method for cell type enrichment analysis based on ssGSEA, to infer the immune score, stromal score, and microenvironment score.

### Immunohistochemistry, Western Blotting and Quantitative Real-Time PCR

The methods for IHC staining, IHC scoring, western blotting and qRT-PCR were described in previous studies (21). Primary antibodies against IGF2BP2 (1:250, D4R2F, Cell Signaling Technology, USA) were utilized for IHC staining, and those against β-actin (1:1,000, D6A8, Cell Signaling Technology, USA) and IGF2BP2 (1:1000, D4R2F, Cell Signaling Technology, USA) were used for western blotting. The primer sequences used were as follow: IGF2BP2-forward: 5′-AGTGGAATTGCATGGGAAAATCA-3′; IGF2BP2- reverse: 5′-CAACGGCGGTTTCTGTGTC-3′; MYC-forward: 5′-TCCCTCCACTCGGAAGGAC-3′; MYC-reverse: 5′-CTGGTGCATTTTCGGTTGTTG-3′; CD8A-forward: 5′-TCCTCCTATACCTCTCCCAAAAC-3′; CD8A-reverse: 5′-GGAAGACCGGCACGAAGTG-3′; β-actin -forward: 5′-CTACCTCATGAAGATCCTCACCGA-3′; and β-actin-reverse: 5′-TTCTCCTTAATGTCACGCACGATT-3′.

### SiRNA Transfection

SCC25 and CAL27 cells were seeded into a 6-well plate and cultured for 24 h, and then PepMute Transfection Reagent (SL100566, Signagen, USA) was applied to transfect 30 nM negative control (siNC) or IGF2BP2 siRNAs (si-IGF2BP2-1: GGGUAGAUAUCCAUAGAAA; si-IGF2BP2-2: AGAUAGAGAUUAUGAAGAA; si-IGF2BP2-3: GUUGAUUACUCAGUCUCUA) per well according to the manufacturer’s instructions. Cells were collected 48 h after transfection.

### Cell Proliferation

SCC25 and CAL27 cells were transfected with siRNA and seeded into 96-well plates at 2,000 cells per well. The time of 0 h was defined as the point were fully attached and Cell Counting Kit-8 (CCK-8, 40203ES80, Yeasen, China) reagent was used to detect cell proliferation at 5 time points (0, 24, 48, 72 and 96 h). One hundred microliters of 10% CCK-8 reagent (10 μL of CCK-8 and 90 μL of serum-free media) were added to each well after aspirating the old media, and the mixture was incubated at 37°C for 1 h. We used a microplate reader (Bio-Rad, USA) to measure the absorbance values at 450 nm and drew a growth curve based on the absorbance values and time.

The colony formation assay was performed by inoculating 500 SCC25 cells or 10,000 CAL 27 cells transfected with siRNA in 6-well plates. After 7-14 days of culture, the cells were stained and fixed. The number of SCC25 cell colonies was directly counted, while the colony number of CAL27 cells was counted in three random fields under a microscope at 5× magnification.

### Migration and Invasion Assays

OSCC cell migration and invasion were estimated by Transwell assays. In short, 200 μL of FBS-free media containing 1.5 × 10^5^ SCC25 cells or 2 × 10^5^ CAL27 cells was plated on the upper chambers of Transwell inserts (for the migration assay, 8-μm pore size, Corning, USA), and Transwell inserts coated with 1 mg/mL Matrigel (for the invasion assay, 354234, Corning, USA), while 800 μL of complete media were added to the lower chambers. After culture for 48 h, cells in the upper chamber were removed, while the cells in the lower chamber were fixed, stained, and counted under a microscope at 100× magnification according to the number of nuclei.

### Statistical Analysis

All statistical analyses were performed using R software (R version 4.1.0, https://www.r-project.org/) or GraphPad Prism 9.0 software. Each *in vitro* experiment was repeated at least three times, and all data are presented as the mean and SD. The normality of the distribution of the data was assessed by the Shapiro-Wilk test. Two-tailed unpaired or paired Student’s t test was used to analyze the significance of differences between the two groups in accordance with the normal distribution, while the Mann-Whitney U test was performed for data with a nonnormal distribution. For the comparisons of three or more groups, one-way or two-way ANOVA was used for parametric analysis, and Kruskal-Wallis tests were performed for nonparametric analysis. The correlation between genes was analyzed using Pearson’s correlation. Differences were considered to be statistically significant at a P value < 0.05.

## Results

### Construction of a Weighted Gene Coexpression Network and Identification of Hub Modules

**Figure 1:**
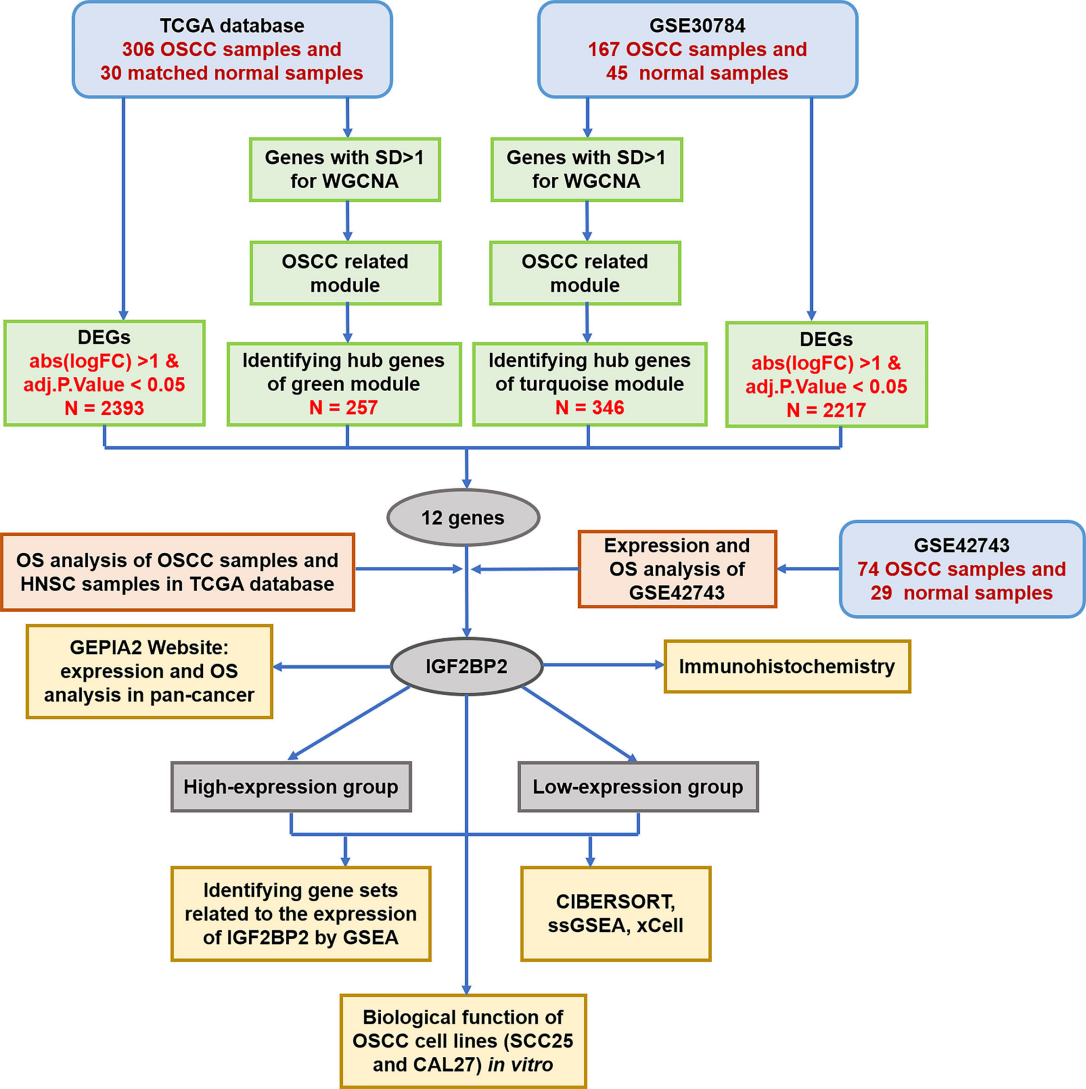
Flowchart of the data preparation, screening, analysis and validation. TCGA, The Cancer Genome Atlas; OSCC, oral squamous cell carcinoma; SD, standard deviation; WGCNA, weighted gene coexpression network analysis; DEGs, differentially expressed genes; OS, overall survival; HNSC, head and neck squamous cell carcinoma; IGF2BP2, insulin-like growth factor 2 mRNA binding protein 2; GEPIA, gene expression profiling interactive analysis; GSEA, gene set enrichment analysis; ssGSEA, single-sample gene set enrichment analysis.

**Figure 2:**
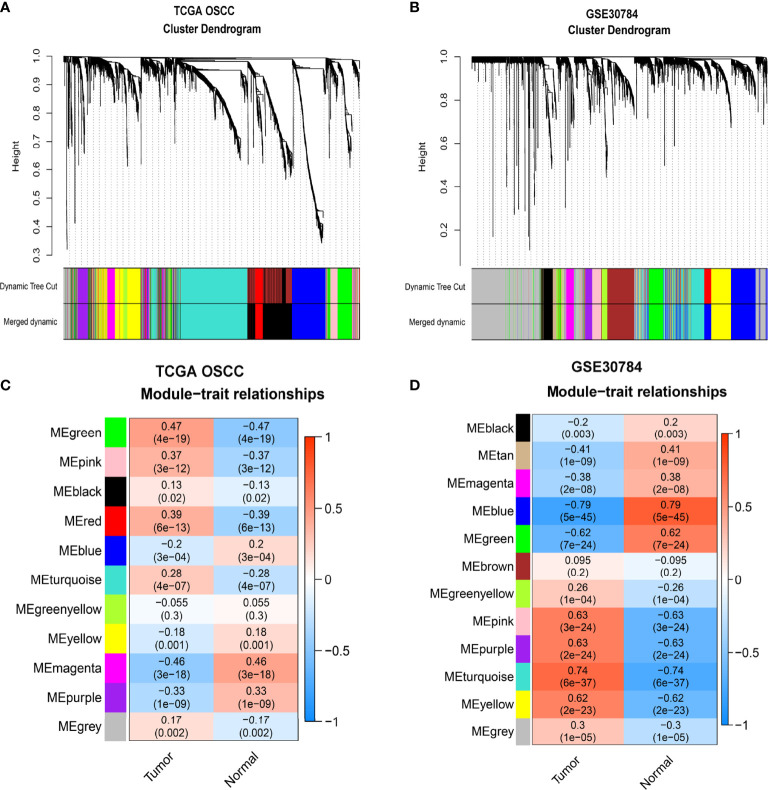
Identification of modules related to OSCC. **(A, B)** Hierarchical clustering tree developed by gene dissimilarity based on topological overlap for TCGA-OSCC **(A)** and GSE30784 datasets **(B)**. Each color represents a module (gray represents unassigned genes). **(C, D)** The correlation coefficients and p-values of module-trait relationships for TCGA-OSCC **(C)** and GSE30784 datasets **(D)**. Each row corresponds to a module eigengene, and each column corresponds to a trait.

**Figure 1** We used TCGA-OSCC and GSE30784 datasets to construct a weighted gene coexpression network and screened the hub module related to the progression of OSCC. First, we filtered and eliminated outliers with the hclust function and drew clustering dendrograms for the remaining samples with a sample type heatmap (**Supplementary Figures 1A, B**). Next, we selected a soft threshold power of 5 for TCGA-OSCC dataset and 11 for the GSE30784 dataset to construct a weighted gene coexpression network based on a scale-free topology fit index reaching 0.85 (**Supplementary Figures 1C, D**). We further assessed the scale-free topology fit index and found that the correlation coefficients (R-squared) were 0.97 and 0.84, respectively, indicating that the selected β values established scale-free networks (**Supplementary Figures 1E, F**). After determining the soft threshold powers, the degree of dissimilarity between genes was calculated by obtaining the adjacency matrix and the topological overlap matrix (TOM). The hierarchical clustering tree was divided into multiple modules (12 modules for TCGA-OSCC dataset and 13 modules for the GSE30784 dataset) using the dynamic tree cut method, and the modules with dissimilarity of <0.2 were merged (**Supplementary Figures 1G, H** and **Figures 2A, B**). We identified 11 modules in TCGA-OSCC dataset, and the module most significantly related to OSCC was the green module (**Figure 2C**), which contained 257 genes (**Supplementary Data Sheet 1**). We identified 12 modules in the GSE30784 dataset, and the module most significantly associated with OSCC was the turquoise module (**Figure 2D**), which contained 346 genes (**Supplementary Data Sheet 2**).

### IGF2BP2 Was Selected as a Hub Gene Through a Prognostic Analysis

**Figure 3:**
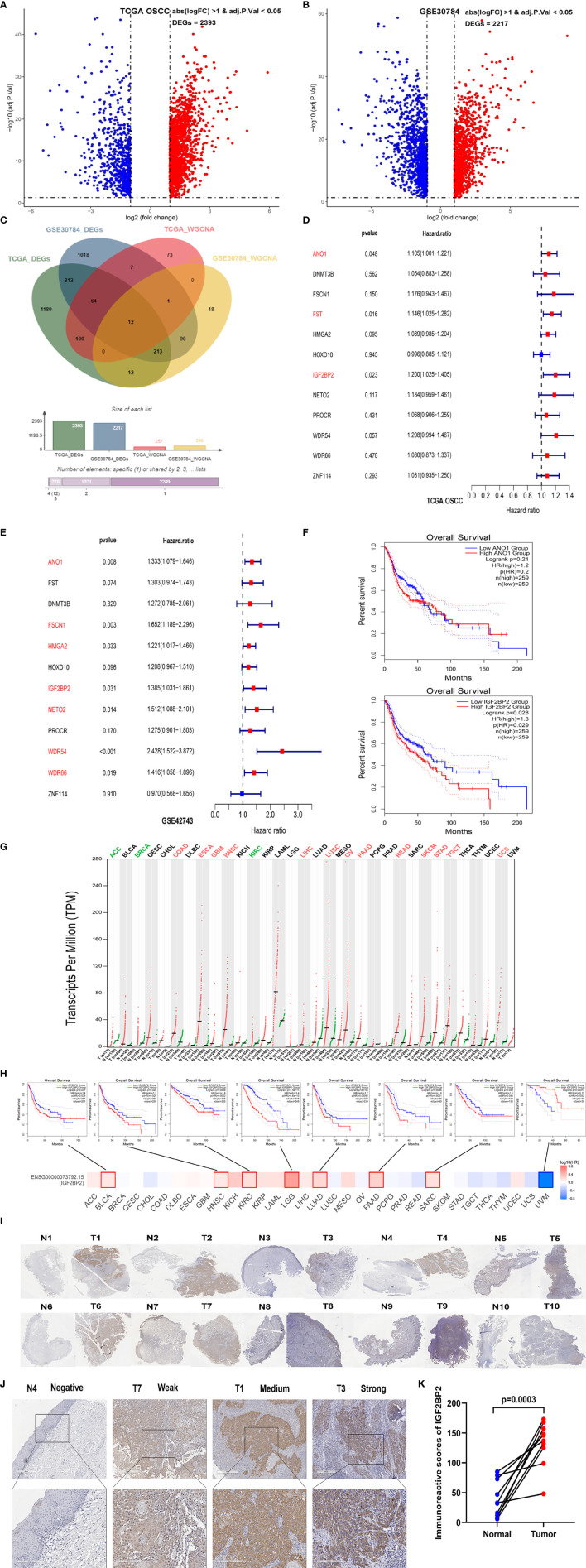
IGF2BP2 was selected as a hub gene through prognostic analysis. **(A)** Volcano plot of 2,393 DEGs between normal (N=30) and OSCC (N=306) tissues from the TCGA database. **(B)** Volcano plot of 2,217 DEGs between OSCC and normal tissues from the GSE30784 dataset. **(C)** The Venn diagram shows 12 common genes of DEGs and hub genes from WGCNA between OSCC tissues and normal tissues in the TCGA and GSE30784 datasets. **(D, E)** The forest plots show the hazard ratios and 95% confidence intervals of 12 hub genes associated with OS in TCGA-OSCC **(D)** and GSE42743 datasets **(E)** according to univariate Cox regression analysis. **(F)** Kaplan–Meier curves of OS based on HNSC patients in the TCGA database with high and low ANO1 and IGF2BP2 expression. **(G)** Differential expression of IGF2BP2 in 33 different tumor tissues and paired normal tissues from the TCGA and GTEx databases. Each dot represents the expression of samples. **(H)** The prognostic impact of IGF2BP2 expression level based on the survival heatmap, showing significance in BLCA, HNSC, KIRC, LGG, LUAD, PAAD, SARC, and UVM. **(I)** Images of IHC staining for IGF2BP2 in 10 pairs of OSCC tissues (T) and adjacent noncancerous normal tissues (N). Magnification at 50× **(J)** Representative images of IHC staining for IGF2BP2 in adjacent noncancerous normal tissues and OSCC tissues. Magnification at 200× **(K)** Histological scoring of IGF2BP2 in 10 pairs of OSCC tissues and adjacent noncancerous normal tissues.

By comparing the expression profiles of OSCC and normal samples, we identified2,393 DEGs in TCGA-OSCC dataset and 2,217 DEGs in the GSE30784 dataset (**Figures 3A, B** and **Supplementary Data Sheets 3**, **4**). In this study, we intersected the genes in the modules most relevant to OSCC and DEGs to intersect and obtained 12 candidate genes between the two datasets (**Figure 3C**). The 12 candidate genes were ANO1, DNMT3B, FSCN1, FST, HMGA2, HOXD10, IGF2BP2, NETO2, PROCR, WDR54, WDR66, and ZNF144. We divided patients into high- and low-expression groups based on the median expression levels of these 12 candidate genes to verify their effects on the prognosis of patients with OSCC. Three candidate genes were found to be significantly associated with the OS of the patients in TCGA-OSCC dataset by applying univariate Cox regression analysis (**Figure 3D**). Seven candidate genes were identified to be significantly related to the OS of the patients in the GSE42743 dataset (**Figure 3E**). Among them, only ANO1 and IGF2BP2 had statistically significantly associated with the OS of patients with OSCC in both datasets. However, ANO1 and IGF2BP2 were not statistically significant with the RFS of patients in the TCGA-OSCC dataset (**Supplementary Figures 2A, B**). We further analyzed the differences in the expression of ANO1 and IGF2BP2 between OSCC samples and normal samples and found higher expression of both ANO1 and IGF2BP2 in OSCC samples than in normal samples in TCGA-OSCC dataset (**Supplementary Figures 2C, D**). The same results were obtained from the GSE30784 and GSE42743 datasets, and but the difference in IGF2BP2 expression was greater than that of ANO1 (**Supplementary Figures 2E–H**). Next, the effects of ANO1 and IGF2BP2 on the prognosis of HNSC patients were evaluated. The GEPIA2 results showed that HNSC patients with high IGF2BP2 expression had a worse OS rate than those with low IGF2BP2 expression, but this pattern was not observed for ANO1 (**Figure 3F**). To understand the expression and prognosis of ANO1 and IGF2BP2 across cancers, we used the GEPIA2 web server to analyze data containing 33 cancer subtypes derived from TCGA and GTEx databases. Compared with that in normal samples, ANO1 expression was significantly reduced in KIRP, PRAD, SKCM, TGCT, UCEC and UCS cancer samples, while it was significantly upregulated in ESCA, HNSC, KIRC, OV, PAAD, STAD and THYM cancer samples (**Supplementary Figure 2I**). IGF2BP2 expression was significantly reduced in ACC, BRCA and KIRC cancer samples, while it was significantly upregulated in COAD, ESCA, GBM, HNSC, LIHC, LUSC, OV, PAAD, READ, SKCM, STAD, TGCT and UCS cancer samples (**Figure 3G**). For the OS analysis, the Kaplan–Meier curves revealed that high ANO1 expression predicted worse prognosis for patients with LIRP, PAAD and UVM (**Supplementary Figure 2J**). While high IGF2BP2 expression predicted worse prognosis for patients with BLCA, HNSC, KIRC, LGG, LUAD, PAAD, SARC, and UVM (**Figure 3H**). Based on the above results, IGF2BP2 was selected as the hub gene for further analysis. We performed IHC staining on 10 pairs of OSCC tissues and adjacent noncancerous normal tissues, and found that the level of the IGF2BP2 protein was also significantly upregulated in OSCC tissues (**Figures 3I–K**).

### Functional Annotation of IGF2BP2 in OSCC

**Figure 4:**
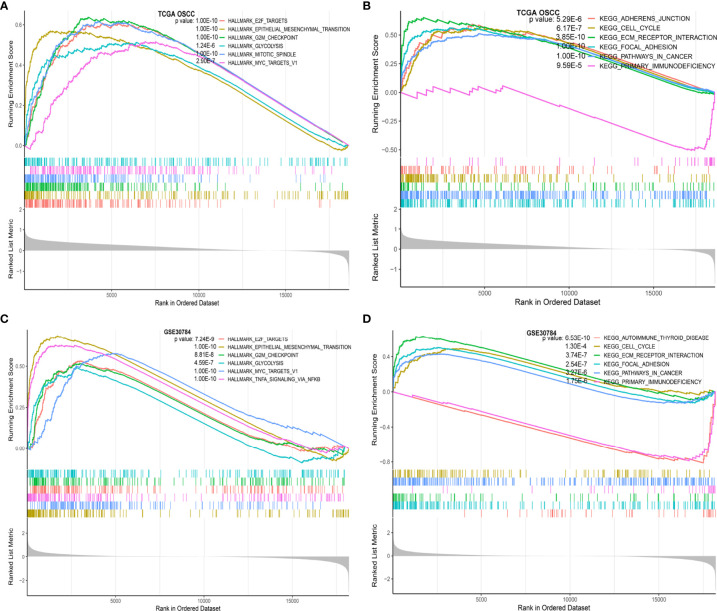
Functional annotation of IGF2BP2 in OSCC. **(A)** The GSEA results show the functional enrichment of hallmark gene sets based on IGF2BP2 expression in TCGA-OSCC dataset; **(B)** GSEA results show the functional enrichment of KEGG gene sets based on IGF2BP2 expression in TCGA-OSCC dataset; **(C)** GSEA results show the functional enrichment of hallmark gene sets based on IGF2BP2 expression in the GSE30784 dataset; **(D)** GSEA results show the functional enrichment of KEGG gene sets based on IGF2BP2 expression in the GSE30784 dataset.

We used the R package “clusterProfiler” to perform GSEA between the high and low IGF2BP2 expression groups in TCGA-OSCC and GSE30784 datasets, respectively (22). Twenty-three enriched gene sets with statistically significant differences (adjusted P value < 0.05, FDR < 0.05) for TCGA-OSCC dataset and 26 enriched gene sets for the GSE30784 dataset in the hallmark gene sets (**Supplementary Data Sheets 5**, **7**) (23). In these two datasets, the hallmark gene sets enriched in the IGF2BP2 high-expression group were mainly “E2F targets”, “epithelial-mesenchymal transition” (EMT), “G2M checkpoint”, “glycolysis”, and “myc targets v1” (**Figures 4A, C**). Thirty-eight enriched gene sets with statistically significant differences from TCGA-OSCC dataset and 39 enriched gene sets from the GSE30784 dataset in the KEGG analysis (**Supplementary Data Sheets 6**, **8**). “Cell cycle”, “ECM receptor interaction”, “focal adhesion”, and “pathways in cancer” were the enriched KEGG gene sets in the high IGF2BP2 expression group, whereas “primary immunodeficiency” was enriched in the low IGF2BP2 expression group (**Figures 4B, D**). Based on the GSEA results, IGF2BP2 might promote the malignant progression of OSCC by affecting cancer-related and immune-related biological functions and pathways.

### Correlation Between Immune Cell Infiltrates and IGF2BP2 Expression in OSCC

**Figure 5:**
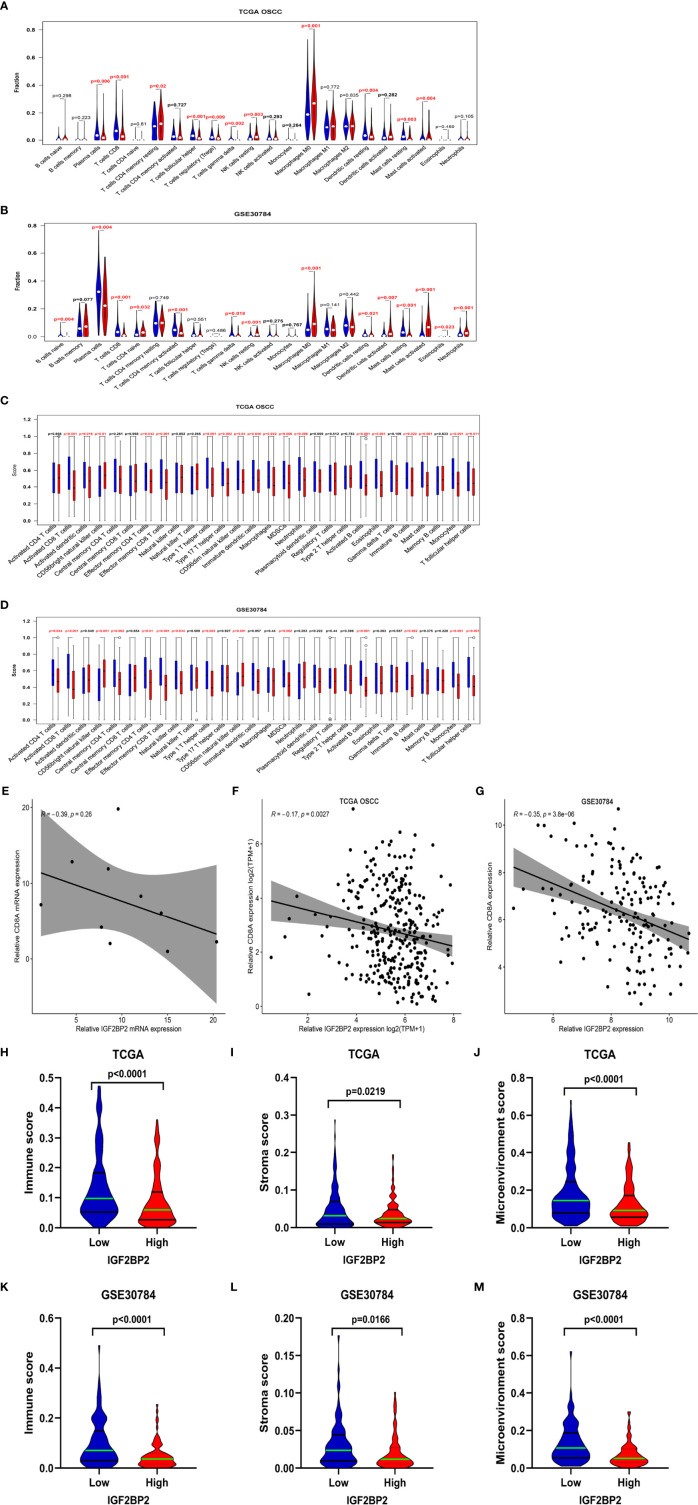
Correlation between immune infiltrates and IGF2BP2 expression in OSCC. **(A, B)** Infiltration fraction between IGF2BP2 expression and 22 immune cells in the OSCC in TCGA-OSCC **(A)** and GSE30784 datasets **(B)** according to the CIBERSORT algorism. **(C, D)** Boxplots show IGF2BP2 expression and the score of 28 immune cells in the OSCC in TCGA-OSCC **(C)** and the GSE30784 datasets **(D)** with ssGSEA algorism. **(E)** Correlation between IGF2BP2 and CD8A expression in TCGA-OSCC dataset. **(F)** Correlation between IGF2BP2 and CD8A expression in GSE30784 dataset. **(G)** Correlation between the mRNA levels of IGF2BP2 and CD8A in 10 OSCC tissues. **(H–J)** The immune score **(H)**, stromal score **(I)**, and microenvironment score **(J)** of the IGF2BP2 high- and low-expression groups in the OSCC in the TCGA database according to the xCell algorithm. **(K–M)** The immune score **(K)**, stromal score **(L)**, and microenvironment score **(M)** of IGF2BP2 high- and low-expression groups in the OSCC in the GSE30784 dataset according to the xCell algorithm.

Immune cell infiltration in the tumor microenvironment (TME) has been shown to play a key role in tumor development and will affect the prognosis of patients with cancer (24). Therefore, we analyzed the correlations between IGF2BP2 expression and infiltrating immune cells in OSCC. The results estimated with the CIBERSORT algorithm showed lower levels of infiltrating plasma cells, CD8 T cells, gamma delta T cells, resting dendritic cells, and resting mast cells (P < 0.05) in the high IGF2BP2 expression group of TCGA-OSCC and GSE30784 cohorts, while resting NK cells, M0 macrophages, and eosinophils (P < 0.05) exhibited higher levels of infiltration (**Figures 5A, B**). Furthermore, ssGSEA revealed that activated CD8 T cells, effector memory CD4 cells, effector memory CD4 cells, type 1 T helper cells, MDSCs, activated B cells, immature B cells, monocytes, and T follicular helper cells (P < 0.05) were significantly less enriched in the high IGF2BP2 expression group of TCGA-OSCC and GSE30784 cohorts. However, the infiltration of CD56 bright natural killer cells (P < 0.05) was positively correlated with IGF2BP2 expression (**Figures 5C, D**). Based on the results obtained from CIBERSORT and ssGSEA, we found that the expression of IGF2BP2 was significantly negatively correlated with the infiltration of immune cells, especially CD8+ T cells. The same results were obtained in from TCGA-OSCC and GSE30784 datasets that IGF2BP2 expression was significantly negatively correlated with the CD8A (A marker gene for CD8+ T cells) (**Figures 5E, F**). RT-qPCR analysis of mRNA levels in 10 OSCC tumor tissues revealed that the mRNA levels of IGF2BP2 was also negatively correlated with that of CD8A (**Figure 5G**). Similarly, the immune score (P < 0.0001), stromal score (P < 0.05), and microenvironment score (P < 0.0001) of the high IGF2BP2 expression group in these two datasets were significantly lower than those of the low IGF2BP2 expression group according to the xCell algorithm (**Figures 5H–M**).

### Silencing IGF2BP2 Inhibits the Proliferation, Migration, and Invasion of OSCC Cells *In Vitro*

**Figure 6:**
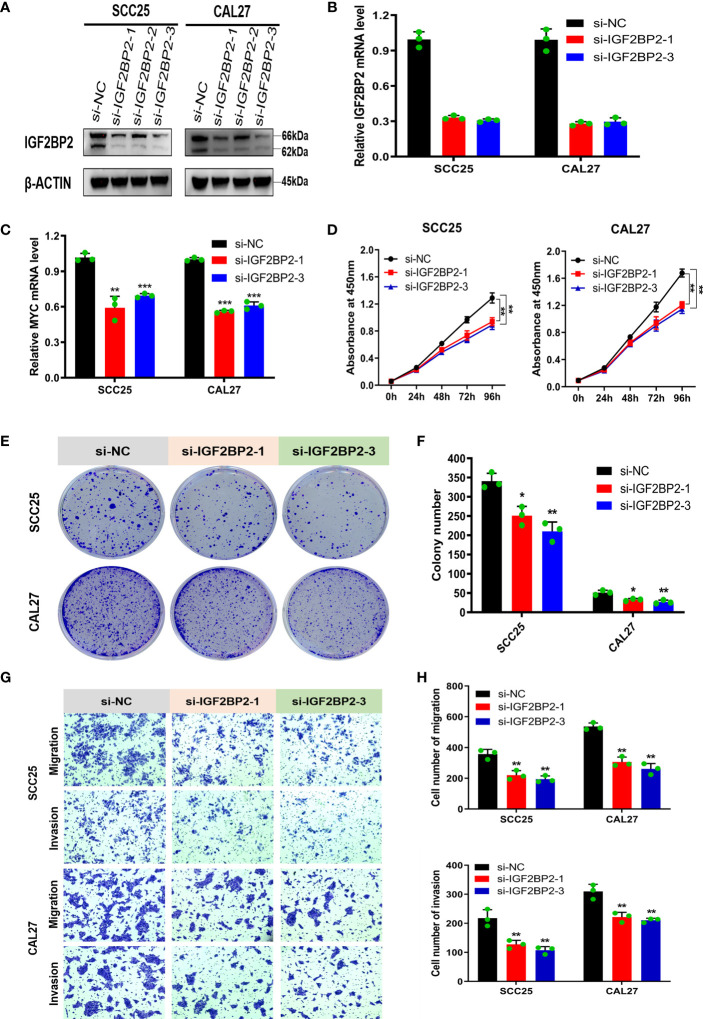
Silencing IGF2BP2 inhibits the proliferation, migration, and invasion of OSCC cells *in vitro*. **(A, B)** Western blotting and qRT-PCR were performed to detect IGF2BP2 expression after transfection of siRNA. **(C)** The mRNA levels of MYC was detected by qRT-PCR in IGF2BP2-knockdown SCC25 and CAL27 cells. **(D–F)** The proliferation of NC and IGF2BP2-silenced SCC25 and CAL27 cells was detected using CCK-8 **(D)** and colony formation assays **(E, F)**. **(G, H)** Transwell assays were performed to assess migration and invasion in NC and IGF2BP2 knockdown SCC25 and CAL27 cells, which were photographed **(G)** and assessed **(H)**; Magnification at 100×. The experiment was repeated three times; error bars indicate standard deviation. *p < 0.05, **p < 0.01, ***p < 0.001.

This study revealed that IGF2BP2 is upregulated in OSCC and is associated with a poor prognosis in patients. The biological function of IGF2BP2 in OSCC cells is still unclear, and we then knocked down IGF2BP2 in SCC25 and CAL27 cells to explore its effects on cell proliferation, migration and invasion *in vitro* (**Figures 6A, B**). We chose two sequences with higher silencing efficiency, si-IGF2BP2-1 and si-IGF2BP2-3, for the subsequent experiments. Given that MYC is a well-defined oncogene, and IGF2BP2 can increase its stability in a m6A-dependent manner. We hypothesized that IGF2BP2 might play an oncogenic role in OSCC. In fact, knockdown of IGF2BP2 in SCC25 and CAL27 cells significantly suppressed MYC expression (**Figure 6C**). The results of correlation analysis also showed that the expressions of IGF2BP2 and MYC were significantly positively correlated in TCGA-OSCC and GSE30784 datasets (**Supplementary Figures 2A, B**). Furthermore, as shown in **Figures 6D–F**, compared with the si-NC group, the IGF2BP2 silencing group showed inhibited proliferation of SCC25 and CAL27 cells according to CCK-8 and colony formation assays. Moreover, the Transwell assay showed that silencing IGF2BP2 suppressed the migration and invasion of SCC25 and CAL27 cells (**Figures 6G, H**). The above results indicated that IGF2BP2 may play an important role in regulating the proliferation, migration and invasion of OSCC cells *in vitro*.

## Discussion

The occurrence and development of OSCC is a complex, multistep, and multifactorial process that mainly includes the dysregulation of oncogenes or tumor suppressor genes, the accumulation of epigenetic changes, and the interaction between tumor cells and the microenvironment (25). OSCC is a severely teratogenic and fatal disease. It is often invasive and accompanied by cervical lymph node metastasis and distant metastasis, which indicates a poor prognosis (26). Therefore, exploring the biological markers of OSCC plays an important role in early diagnosis and improvement of prognosis. We performed WGCNA to screen the key modules in TCGA-OSCC and GSE30784 gene sets, intersected the genes in the modules with DEGs, and then performed hub gene selection and survival analysis. Finally, IGF2BP2 was selected as the hub gene and as a biomarker that affects the prognosis of OSCC. The results of GSEA and tumor-infiltrating immune cell analysis indicated that IGF2BP2 might promote the malignant progression of OSCC by affecting cancer-related and immune-related biological functions and pathways. Moreover, silencing IGF2BP2 inhibited the proliferation, migration and invasion of SCC25 and CAL27 cells *in vitro*. The present study revealed that IGF2BP2 may act as a prognostic and immune biomarker by promoting the proliferation, migration and invasion of OSCC cells.

Several independent datasets indicated that IGF2BP2 was expressed at high levels in OSCC tissues and that high expression predicts a worse prognosis than low expression, consistent with studies on acute myelocytic leukemia (27), hepatocellular carcinoma (11), and pancreatic cancer (28). The results of the pan-cancer analysis also showed that IGF2BP2 was upregulated in a variety of cancers and was negatively correlated with the OS rate. In addition, our IHC results also showed that IGF2BP2 was expressed at high levels in OSCC tissues, indicating that IGF2BP2 functions as an oncogene in OSCC.

We conducted a GSEA to identify gene sets related to IGF2BP2 expression and to explore the molecular mechanism by which IGF2BP2 expression contributes to the malignant progression of OSCC using OSCC expression data provided by TCGA-OSCC and GSE30784 datasets. According to the GSEA results, high expression of IGF2BP2 mainly activates the EMT, glycolysis, and cell cycle three cancer-related pathways that affect cancer cell proliferation, migration, and invasion. The regulatory effect of IGF2BP2 on cancer cell proliferation, migration, and invasion is an important factor contributing to prognosis. Recently, LINC01559 was found to recruit IGF2BP2 to stabilize ZEB1 expression and accelerate gastric cancer cell proliferation, migration and EMT (29). A study on GBM found that by activating the IGF2BP2/PI3K/Akt axis, IGF2BP2 can significantly promote cell proliferation, migration, invasion, and EMT (30). Additionally, long noncoding RNA (LncRNA) CASC9 and IGF2BP2 synergistically increases the stability of HK2 mRNA, thereby promoting aerobic glycolysis in GBM (31). Similarly, IGF2BP2 stabilizes the HK2 and SLC2A1 mRNAs, and accelerates glycolytic metabolism and cell proliferation in colorectal cancer (32). This evidence indicates that IGF2BP2 promotes the proliferation, migration and invasion of cancer cells. Consistently, the results of this study also demonstrate that IGF2BP2 knockdown inhibits the proliferation, migration and invasion of OSCC cells *in vitro*. In summary, IGF2BP2 may facilitate the malignant progression of OSCC by enhancing glycolysis, inducing EMT, and promoting the cell cycle.

Antitumor immunotherapy is based on the principle that immune monitoring and the adaptability of the TME allow immune escape (33). Immune cells are the cellular basis of antitumor immunotherapy. A comprehensive analysis of tumor-infiltrating immune cells will help clarify the mechanism of tumor immune evasion, which is the key to improving the response rate of immunotherapy and developing new treatment strategies (24, 34). The imbalance of the TME may also be an important reason IGF2BP2 affects the progression and confers a poor prognosis in OSCC. As shown in the present study, high IGF2BP2 expression was accompanied by a significant reduction in the immune score, stromal score, and microenvironment score and a decrease in infiltrating CD8+ T cells. CD8+ T cells play a central role in tumor immunity and can specifically kill tumor cells. Reduced infiltration or impaired function of CD8+ T cells in the TME can lead to a poor prognosis for many cancers (35). Shimizu et al. showed that in the invading edge and peripheral stroma of OSCC, an increase in tumor infiltrating CD8+ T cells was associated with an improvement in OS and disease-specific survival (36). Tabachnyk et al. found that the increase in tumor-infiltrating CD8+ T cells in OSCC patients significantly increased disease-free survival after concurrent radiotherapy and chemotherapy (36). However, programmed cell death-ligand 1 (PD-L1) on the surface of tumor cells binds to the activated CD8+ T cell receptor programmed cell death-1 (PD-1), which significantly inhibits the ability of CD8+ T cells to kill tumor cells. The effect of anti-PD-1 monoclonal antibody treatment can be reflected in the density of tumor-infiltrating CD8+ T cells in solid tumors (37). A recent study suggested that IGF2BP2 can combine with PD-L1 to promote the proliferation and inhibit the apoptosis of hypopharyngeal carcinoma cells through the PD-1/PD-L1 axis (38). Taken together, these data indicate that IGF2BP2 may affect the infiltration of CD8+ T cells in OSCC and is related to the efficacy of antitumor immunotherapy response and the prognosis, but these findings require further confirmation.

Multiple mechanisms may account for the role of IGF2BP2 in tumor progression. IGF2BP2 is a posttranscriptional regulator of mRNA localization, stabilization and translation and is related to the regulation of the expression of miRNAs, lncRNAs and m6A-related genes (39). Target binding of miRNAs to IGF2BP2 inhibits its expression and malignant tumor progression. For example, miR-1193 activates ERK and PI3K/Akt signaling pathways by binding to the 3’ UTR of the IGF2BP2 mRNA to inhibit the proliferation and invasion of breast cancer cells (40). In addition, miR-138 inhibitsIGF2BP2 expression by targeting its 3′-UTR, thereby inhibiting the EMT and reducing the proliferation and invasion of low-grade glioma cells (41). IGF2BP2 interacts with lncRNAs to maintain multiple malignant biological behaviors of tumors. For instance, the lncRNA LINRIS blocks the ubiquitination of K139 in IGF2BP2 and prevents its degradation through the autophagy-lysosomal pathway, thus maintaining MYC-mediated glycolysis and promoting the proliferation of colorectal cancer cells (42). Moreover, linc01305 increases the stability of HTR3A mRNA by interacting with IGF2BP2, thereby promoting the metastasis and proliferation of esophageal squamous cell carcinoma (43). A recent study found that IGF2BP2 is an m6A-binding protein that enhances the stability of m6A-related genes/mRNAs and promotes translation (10). Li et al. proved that METTL3 upregulates SOX2 expression through IGF2BP2 to recognize and increase the stability of SOX2 mRNA and promote colorectal carcinoma cell self-renewal, increase stem cell frequency and promote migration in a m6A-dependent manner (44). In hepatocellular carcinoma, IGF2BP2 directly recognizes and binds to the m6A site of the FEN1 mRNA, increased the stability of FEN1 mRNA, and promotes the proliferation of hepatocellular carcinoma cells (11). At present, the mechanisms by which IGF2BP2 promotes the proliferation and metastasis of OSCC cells are still unclear. However, the mechanisms may involve the EMT, glycolysis, and tumor infiltrating immune cells, and further research is needed.

In summary, we conducted WGCNA to identify the key modules and genes related to OSCC that intersect with DEGs and performed an analysis of the survival prognosis and IHC to screen and validate the hub gene IGF2BP2. The GSEA results showed that IGF2BP2 promotes the malignant progression of OSCC by affecting cancer-related processes (EMT, glycolysis, cell cycle, etc.) and immune-related biological functions and pathways. Our study also revealed that IGF2BP2 is closely associated with remodeling immune microenvironment and that high IGF2BP2 expression is accompanied by a decrease in the number of tumor-infiltrating CD8+ T cells. In addition, we demonstrated that IGF2BP2 knockdown inhibits the proliferation, migration and invasion of OSCC cells *in vitro*. However, these findings must be verified *in vivo* in our future studies. Moreover, the molecular mechanism of IGF2BP2 in the progression of OSCC should be further systematically elucidated and investigated in depth.

## Data Availability Statement

The original contributions presented in the study are included in the article/**Supplementary Material**. Further inquiries can be directed to the corresponding authors.

## Ethics Statement

The studies involving human participants were reviewed and approved by the Stomatology Hospital Research Ethics Committee of Sun Yat-sen University. The patients/participants provided their written informed consent to participate in this study.

## Author Contributions

LZ, DY, and SH conceived and designed the study. LZ, WL, and EP performed the data acquisition and analysis. LZ, HL, HC, WL, and EP prepared the figures and supplementary materials. HL and HC performed the experiments. LZ, HL, and HC wrote the original manuscript. DY and SH reviewed and revised the manuscript. All authors read and approved the final manuscript.

## Funding

This study was supported by the National Natural Science Foundation of China (82073378), Natural Science Foundation of Guangdong province (2021A1515012399), and Science and Technology Plan Project of Wenzhou (Y20190446).

## Conflict of Interest

The authors declare that the research was conducted in the absence of any commercial or financial relationships that could be construed as a potential conflict of interest.

## Publisher’s Note

All claims expressed in this article are solely those of the authors and do not necessarily represent those of their affiliated organizations, or those of the publisher, the editors and the reviewers. Any product that may be evaluated in this article, or claim that may be made by its manufacturer, is not guaranteed or endorsed by the publisher.

## Supplementary Material

Supplementary Data Sheet 1 Genes in the green module for TCGA-OSCC dataset.

Supplementary Data Sheet 2 Genes in the turquoise module for the GSE30784 dataset.

Supplementary Data Sheet 3 DEGs between OSCC and normal samples in TCGA-OSCC dataset.

Supplementary Data Sheet 4 DEGs between OSCC and normal samples in the GSE30784 dataset.

Supplementary Data Sheet 5 Enriched Hallmark gene sets based on IGF2BP2 expression in TCGA-OSCC dataset.

Supplementary Data Sheet 6 Enriched KEGG gene sets based on IGF2BP2 expression in TCGA-OSCC dataset.

Supplementary Data Sheet 7 Enriched Hallmark gene sets based on IGF2BP2 expression in the GSE30784 dataset.

Supplementary Data Sheet 8 Enriched KEGG gene sets based on IGF2BP2 expression in the GSE30784 dataset.

## References

[1] Bosetti C Carioli G Santucci C Bertuccio P Gallus S Garavello W. Global Trends in Oral and Pharyngeal Cancer Incidence and Mortality. Int J Cancer (2020) 147(4):1040– x9. doi: 10.1002/ijc.32871 31953840

[2] Mupparapu M Shanti RM. Evaluation and Staging of Oral Cancer. Dental Clinics North Am (2018) 62(1):47–58. doi: 10.1016/j.cden.2017.08.003 29126493

[3] Sung H Ferlay J Siegel RL Laversanne M Soerjomataram I Jemal A. Global Cancer Statistics 2020: GLOBOCAN Estimates of Incidence and Mortality Worldwide for 36 Cancers in 185 Countries. CA Cancer J Clin (2021) 71(3):209–49. doi: 10.3322/caac.21660 33538338

[4] Zeng H Chen W Zheng R Zhang S Ji JS Zou X. Changing Cancer Survival in China During 2003–15: A Pooled Analysis of 17 Population-Based Cancer Registries. Lancet Global Health (2018) 6(5):e555–67. doi: 10.1016/s2214-109x(18)30127-x 29653628

[5] Langfelder P Horvath S. WGCNA: An R Package for Weighted Correlation Network Analysis. BMC Bioinf (2008) 9:559. doi: 10.1186/1471-2105-9-559 PMC2631488 19114008

[6] Christiansen J Kolte AM Hansen TVO Nielsen FC. IGF2 mRNA-Binding Protein 2: Biological Function and Putative Role in Type 2 Diabetes. J Mol Endocrinol (2009) 43(5-6):187–95. doi: 10.1677/JME-09-0016 19429674

[7] Nielsen J Christiansen J Lykke-Andersen J Johnsen AH Wewer UM Nielsen FC. A Family of Insulin-Like Growth Factor II mRNA-Binding Proteins Represses Translation in Late Development. Mol Cell Biol (1999) 19(2):1262–70. doi: 10.1128/MCB.19.2.1262 PMC116055 9891060

[8] Zhao Y Ma Y-S Fang Y Liu L Wu S-D Fu D. IGF2BP2 Genetic Variation and Type 2 Diabetes: A Global Meta-Analysis. DNA Cell Biol (2012) 31(5):713–20. doi: 10.1089/dna.2011.1400 22032244

[9] Dai N. The Diverse Functions of IMP2/IGF2BP2 in Metabolism. Trends Endocrinol Metab (2020) 31(9):670–9. doi: 10.1016/j.tem.2020.05.007 32586768

[10] Huang H Weng H Sun W Qin X Shi H Wu H. Recognition of RNA N(6)-Methyladenosine by IGF2BP Proteins Enhances mRNA Stability and Translation. Nat Cell Biol (2018) 20(3):285–95. doi: 10.1038/s41556-018-0045-z PMC5826585 29476152

[11] Pu J Wang J Qin Z Wang A Zhang Y Wu X. IGF2BP2 Promotes Liver Cancer Growth Through an M6a-FEN1-Dependent Mechanism. Front Oncol (2020) 10:578816. doi: 10.3389/fonc.2020.578816 33224879 PMC7667992

[12] Deng X Jiang Q Liu Z Chen W. Clinical Significance of an M6a Reader Gene, IGF2BP2, in Head and Neck Squamous Cell Carcinoma. Front Mol Biosci (2020) 7:68. doi: 10.3389/fmolb.2020.00068 32391379 PMC7193208

[13] Chou CH Chang CY Lu HJ Hsin MC Chen MK Huang HC. IGF2BP2 Polymorphisms Are Associated With Clinical Characteristics and Development of Oral Cancer. Int J Mol Sci (2020) 21(16):5662. doi: 10.3390/ijms21165662 PMC7460642 32784624

[14] Goldman M Craft B Hastie M Repečka K Kamath A McDade F. The UCSC Xena Platform for Cancer Genomics Data Visualization and Interpretation. bioRxiv (2018). doi: 10.1101/326470

[15] Ritchie ME Phipson B Wu D Hu Y Law CW Shi W. Limma Powers Differential Expression Analyses for RNA-Sequencing and Microarray Studies. Nucleic Acids Res (2015) 43(7):e47. doi: 10.1093/nar/gkv007 25605792 PMC4402510

[16] Bardou P Mariette J Escudie F Djemiel C Klopp C. Jvenn: An Interactive Venn Diagram Viewer. BMC Bioinf (2014) 15:293. doi: 10.1186/1471-2105-15-293 PMC4261873 25176396

[17] Subramanian A Tamayo P Mootha VK Mukherjee S Ebert BL Gillette MA. Gene Set Enrichment Analysis: A Knowledge-Based Approach for Interpreting Genome-Wide Expression Profiles. Proc Natl Acad Sci USA (2005) 102(43):15545–50. doi: 10.1073/pnas.0506580102 PMC1239896 16199517

[18] Chen B Khodadoust MS Liu CL Newman AM Alizadeh AA. Profiling Tumor Infiltrating Immune Cells With CIBERSORT. Methods Mol Biol (2018) 1711:243–59. doi: 10.1007/978-1-4939-7493-1_12 PMC5895181 29344893

[19] Hanzelmann S Castelo R Guinney J. GSVA: Gene Set Variation Analysis for Microarray and RNA-Seq Data. BMC Bioinf (2013) 14:7. doi: 10.1186/1471-2105-14-7 PMC3618321 23323831

[20] Aran D Hu Z Butte AJ. Xcell: Digitally Portraying the Tissue Cellular Heterogeneity Landscape. Genome Biol (2017) 18(1):220. doi: 10.1186/s13059-017-1349-1 29141660 PMC5688663

[21] Cai H Li J Zhang Y Liao Y Zhu Y Wang C. LDHA Promotes Oral Squamous Cell Carcinoma Progression Through Facilitating Glycolysis and Epithelial-Mesenchymal Transition. Front Oncol (2019) 9:1446. doi: 10.3389/fonc.2019.01446 31921691 PMC6930919

[22] Wu T Hu E Xu S Chen M Guo P Dai Z. Clusterprofiler 4.0: A Universal Enrichment Tool for Interpreting Omics Data. Innovation (N Y) (2021) 2(3):100141. doi: 10.1016/j.xinn.2021.100141 34557778 PMC8454663

[23] Liberzon A Birger C Thorvaldsdottir H Ghandi M Mesirov JP Tamayo P. The Molecular Signatures Database (MSigDB) Hallmark Gene Set Collection. Cell Syst (2015) 1(6):417–25. doi: 10.1016/j.cels.2015.12.004 PMC4707969 26771021

[24] Zhang Y Zhang Z. The History and Advances in Cancer Immunotherapy: Understanding the Characteristics of Tumor-Infiltrating Immune Cells and Their Therapeutic Implications. Cell Mol Immunol (2020) 17(8):807–21. doi: 10.1038/s41423-020-0488-6 PMC7395159 32612154

[25] Li CC Shen Z Bavarian R Yang F Bhattacharya A. Oral Cancer: Genetics and the Role of Precision Medicine. Surg Oncol Clinics North Am (2020) 29(1):127–44. doi: 10.1016/j.soc.2019.08.010 31757309

[26] Bagan J Sarrion G Jimenez Y. Oral Cancer: Clinical Features. Oral Oncol (2010) 46(6):414–7. doi: 10.1016/j.oraloncology.2010.03.009 20400366

[27] He X Li W Liang X Zhu X Zhang L Huang Y. IGF2BP2 Overexpression Indicates Poor Survival in Patients With Acute Myelocytic Leukemia. Cell Physiol Biochem (2018) 51(4):1945–56. doi: 10.1159/000495719 30513526

[28] Dahlem C Barghash A Puchas P Haybaeck J Kessler SM. The Insulin-Like Growth Factor 2 mRNA Binding Protein IMP2/IGF2BP2 Is Overexpressed and Correlates With Poor Survival in Pancreatic Cancer. Int J Mol Sci (2019) 20(13):3204. doi: 10.3390/ijms20133204 PMC6651604 31261900

[29] Shen H Zhu H Chen Y Shen Z Qiu W Qian C. ZEB1-Induced LINC01559 Expedites Cell Proliferation, Migration and EMT Process in Gastric Cancer Through Recruiting IGF2BP2 to Stabilize ZEB1 Expression. Cell Death Dis (2021) 12(4):349. doi: 10.1038/s41419-021-03571-5 33824282 PMC8024305

[30] Mu Q Wang L Yu F Gao H Lei T Li P. Imp2 Regulates GBM Progression by Activating IGF2/PI3K/Akt Pathway. Cancer Biol Ther (2015) 16(4):623–33. doi: 10.1080/15384047.2015.1019185 PMC4622833 25719943

[31] Liu H Qin S Liu C Jiang L Li C Yang J. M(6)A Reader IGF2BP2-Stabilized CASC9 Accelerates Glioblastoma Aerobic Glycolysis by Enhancing HK2 mRNA Stability. Cell Death Discov (2021) 7(1):292. doi: 10.1038/s41420-021-00674-y 34645788 PMC8514511

[32] Shen C Xuan B Yan T Ma Y Xu P Tian X. M(6)A-Dependent Glycolysis Enhances Colorectal Cancer Progression. Mol Cancer (2020) 19(1):72. doi: 10.1186/s12943-020-01190-w 32245489 PMC7118901

[33] Kraehenbuehl L Weng CH Eghbali S Wolchok JD Merghoub T. Enhancing Immunotherapy in Cancer by Targeting Emerging Immunomodulatory Pathways. Nat Rev Clin Oncol (2021) 19(1):37–50. doi: 10.1038/s41571-021-00552-7 34580473

[34] Cramer JD Burtness B Ferris RL. Immunotherapy for Head and Neck Cancer: Recent Advances and Future Directions. Oral Oncol (2019) 99:104460. doi: 10.1016/j.oraloncology.2019.104460 31683169 PMC7749717

[35] Ock CY Keam B Kim S Lee JS Kim M Kim TM. Pan-Cancer Immunogenomic Perspective on the Tumor Microenvironment Based on PD-L1 and CD8 T-Cell Infiltration. Clin Cancer Res: Off J Am Assoc Cancer Res (2016) 22(9):2261–70. doi: 10.1158/1078-0432.CCR-15-2834 26819449

[36] Shimizu S Hiratsuka H Koike K Tsuchihashi K Sonoda T Ogi K. Tumor-Infiltrating CD8(+) T-Cell Density Is an Independent Prognostic Marker for Oral Squamous Cell Carcinoma. Cancer Med (2019) 8(1):80–93. doi: 10.1002/cam4.1889 30600646 PMC6346233

[37] Doroshow DB Bhalla S Beasley MB Sholl LM Kerr KM Gnjatic S. PD-L1 as a Biomarker of Response to Immune-Checkpoint Inhibitors. Nat Rev Clin Oncol (2021) 18(6):345–62. doi: 10.1038/s41571-021-00473-5 33580222

[38] Yang X Liu J. Targeting PD-L1 (Programmed Death-Ligand 1) and Inhibiting the Expression of IGF2BP2 (Insulin-Like Growth Factor 2 mRNA-Binding Protein 2) Affect the Proliferation and Apoptosis of Hypopharyngeal Carcinoma Cells. Bioengineered (2021) 12(1):7755–64. doi: 10.1080/21655979.2021.1983278 PMC8806995 34608837

[39] Wang J Chen L Qiang P. The Role of IGF2BP2, an M6a Reader Gene, in Human Metabolic Diseases and Cancers. Cancer Cell Int (2021) 21(1):99. doi: 10.1186/s12935-021-01799-x 33568150 PMC7876817

[40] Li X Li Y Lu H. miR-1193 Suppresses Proliferation and Invasion of Human Breast Cancer Cells Through Directly Targeting Igf2bp2. Oncol Res (2017) 25(4):579–85. doi: 10.3727/97818823455816X14760504645779 PMC7841109 27733218

[41] Yang Y Liu X Cheng L Li L Wei Z Wang Z. Tumor Suppressor microRNA-138 Suppresses Low-Grade Glioma Development and Metastasis *via* Regulating Igf2bp2. OncoTargets Ther (2020) 13:2247–60. doi: 10.2147/OTT.S232795 PMC7082711 32214825

[42] Wang Y Lu JH Wu QN Jin Y Wang DS Chen YX. LncRNA LINRIS Stabilizes IGF2BP2 and Promotes the Aerobic Glycolysis in Colorectal Cancer. Mol Cancer (2019) 18(1):174. doi: 10.1186/s12943-019-1105-0 31791342 PMC6886219

[43] Huang GW Chen QQ Ma CC Xie LH Gu J. Linc01305 Promotes Metastasis and Proliferation of Esophageal Squamous Cell Carcinoma Through Interacting With IGF2BP2 and IGF2BP3 to Stabilize HTR3A mRNA. Int J Biochem Cell Biol (2021) 136:106015. doi: 10.1016/j.biocel.2021.106015 34022433

[44] Li T Hu PS Zuo Z Lin JF Li X Wu QN. METTL3 Facilitates Tumor Progression *via* an M(6)A-IGF2BP2-Dependent Mechanism in Colorectal Carcinoma. Mol Cancer (2019) 18(1):112. doi: 10.1186/s12943-019-1038-7 31230592 PMC6589893

