# Supplementary material for: Upregulation of IGF2BP2 Promotes Oral Squamous Cell Carcinoma Progression That Is Related to Cell Proliferation, Metastasis and Tumor-Infiltrating Immune Cells
Source: Front Oncol. 2022 Mar 1;12:809589. doi: 10.3389/fonc.2022.809589 (PMC8921988; doi:10.3389/fonc.2022.809589)
Supplement: Supplementary file 9 [file Image_1.pdf]

Supplementary Material

Supplementary Figures

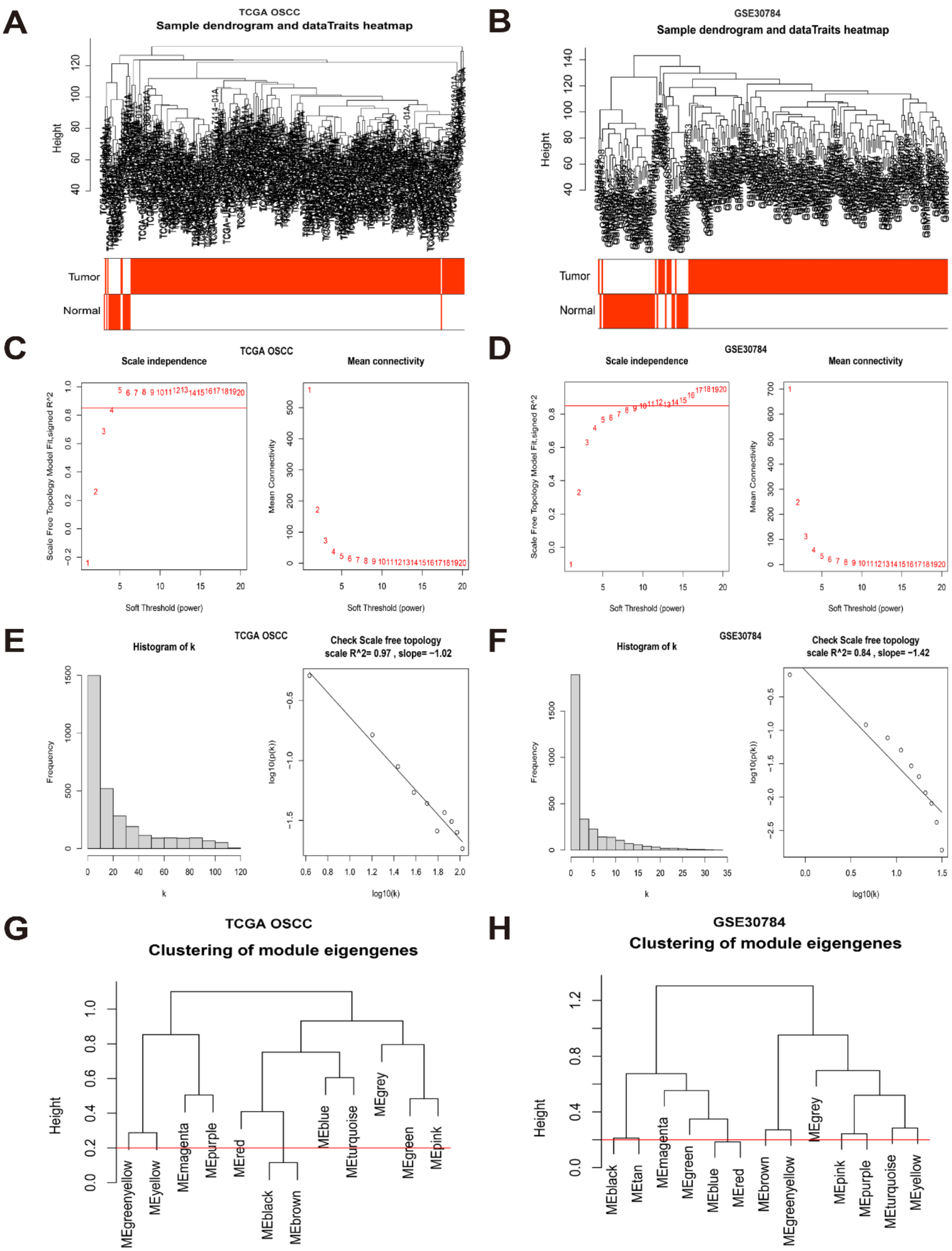

Supplementary Figure 1. Construction of the weighted gene coexpression network. **(A)** Clustering dendrogram for the TCGA-OSCC dataset after removing 11 outliers (N=325). **(B)** Cluster dendrogram for the GSE30784 dataset after removing 3 outliers (N=209). **(C, D)** Analysis of scale-free gene network topology and mean connectivity of various soft-threshold powers for the TCGA-OSCC **(C)** and GSE30784 datasets **(D). (E, F)** Scale-free network topology test based on soft-threshold power for the TCGA-OSCC **(E)** and GSE30784 datasets **(F). (G, H)** Clustering of consensus module eigengenes for the TCGA-OSCC **(G)** and GSE30784 datasets (H); genes with correlations between modules > 0.8, below the red line, were merged.

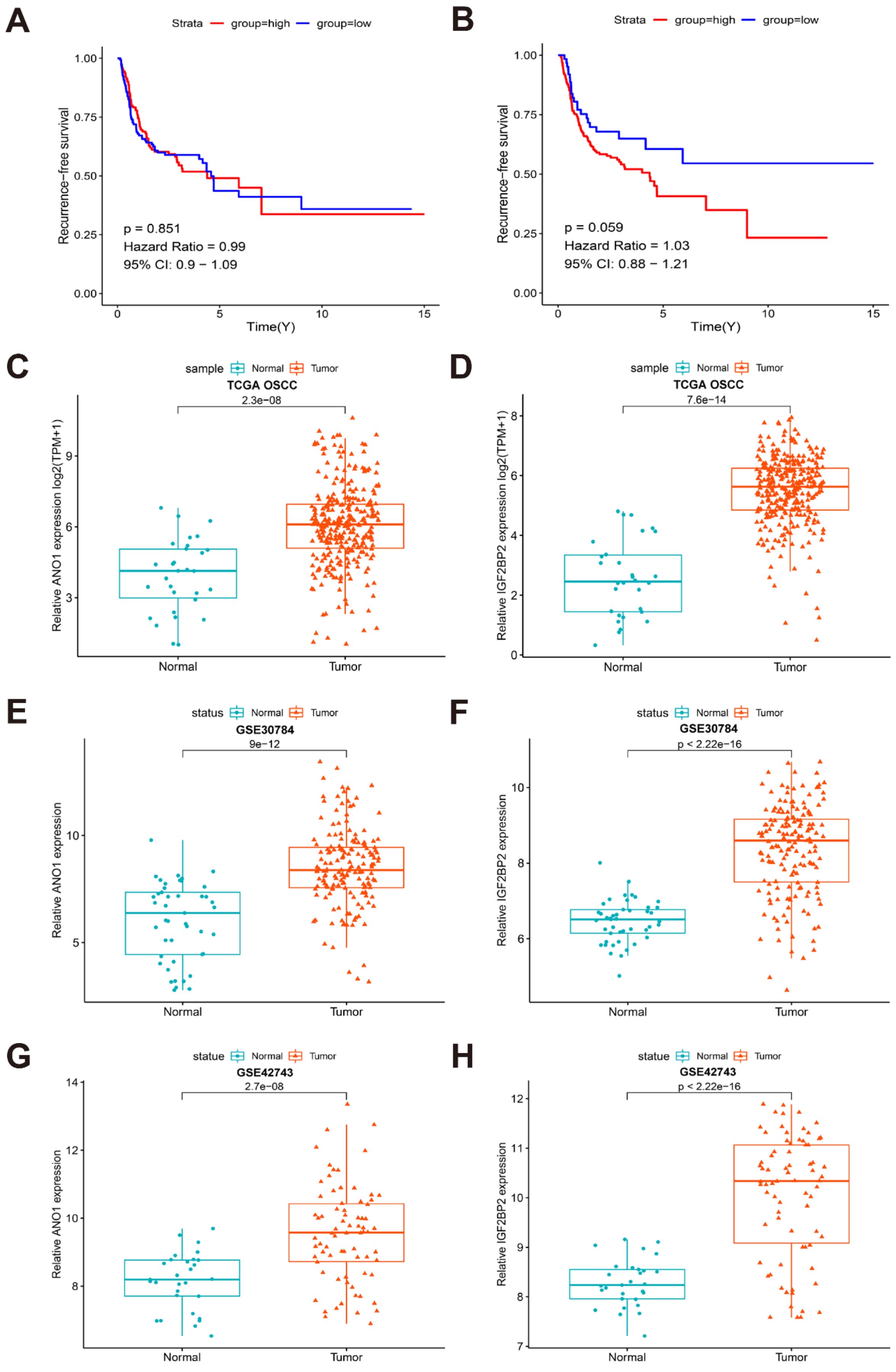

A
Strata group=high group=low
Recurrence−free survival
1.00
0.75
0.50
0.25
0.00
p = 0.851
Hazard Ratio = 0.99
95% CI: 0.9 − 1.09
0
5
10
15
Time(Y)
B
Strata group=high group=low
Recurrence−free survival
p = 0.059
Hazard Ratio = 1.03
95% CI: 0.88 − 1.21
Time(Y)
C
sample Normal Tumor
TCGA OSCC
2.3e−08
Relative ANO1 expression log2(TPM+1)
Normal
Tumor
D
sample Normal Tumor
TCGA OSCC
7.6e−14
Relative IGF2BP2 expression log2(TPM+1)
Normal
Tumor
E
status Normal Tumor
GSE30784
9e−12
Relative ANO1 expression
Normal
Tumor
F
status Normal Tumor
GSE30784
p < 2.22e−16
Relative IGF2BP2 expression
Normal
Tumor
G
statue Normal Tumor
GSE42743
2.7e−08
Relative ANO1 expression
Normal
Tumor
H
statue Normal Tumor
GSE42743
p < 2.22e−16
Relative IGF2BP2 expression
Normal
Tumor

I

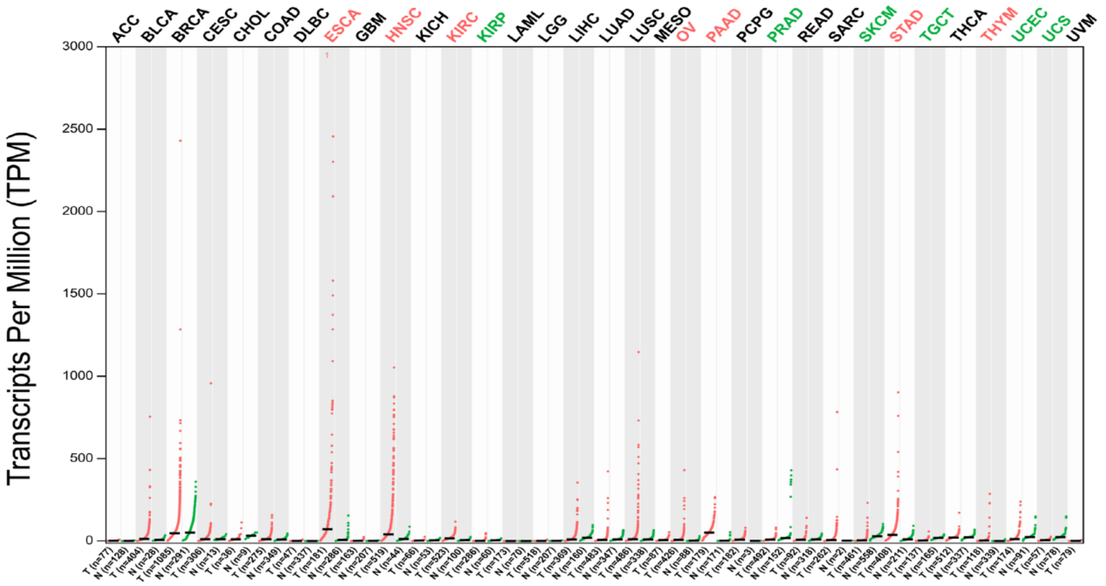

J

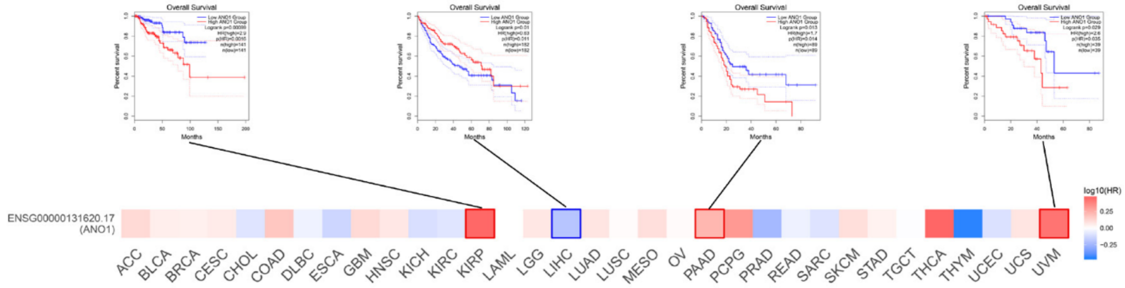

Supplementary Figure 2. Expression and survival analysis of ANO1 and IGF2BP2. **(A, B)** Survival curves of RFS based on OSCC patients in the TCGA database with high and low ANO1 and IGF2BP2 expression. **(C)** ANO1 expression in normal (N=30) and OSCC (N=306) tissues from the TCGA database. **(D)** IGF2BP2 expression in normal (N=30) and OSCC tissues (N=306) from the TCGA database. **(E)** ANO1 expression in normal (N=45) and OSCC tissues (N=167) from the GSE30784 dataset. **(F)** IGF2BP2 expression in normal (N=45) and OSCC tissues (N=167) from the GSE30784 dataset. **(G)** ANO1 expression in normal (N=29) and OSCC tissues (N=74) from the GSE42743 dataset. **(H)** IGF2BP2 expression in normal (N=29) and OSCC tissues (N=74) from the GSE42743 dataset. **(G)** Differential expression of ANO1 in 33 different tumor tissues and paired normal tissues from the TCGA and GTEx databases. Each dot represents the expression of samples. **(H)** The prognostic impact of ANO1 expression level based on the survival heatmap, showing significance in KIRP, LIHC, PAAD, and UVM.

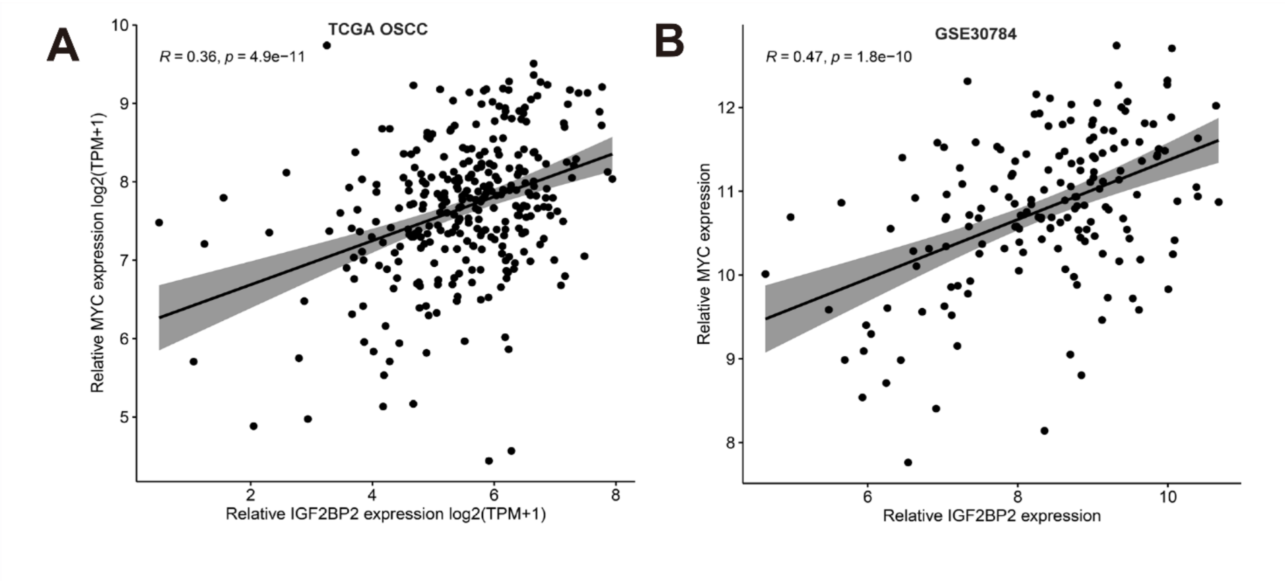

Supplementary Figure 2. Correlation between IGF2BP2 and MYC expression. **(A)** Correlation between IGF2BP2 and CD8A expression in TCGA-OSCC dataset. **(B)** Correlation between IGF2BP2 and MYC expression in GSE30784 dataset.
